# Supplementary figures and images for: Hatching of whipworm eggs induced by bacterial contact is serine-protease dependent
Source: PLoS Pathog. 2025 Jan 27;21(1):e1012502. doi: 10.1371/journal.ppat.1012502 (PMC11819529; doi:10.1371/journal.ppat.1012502)

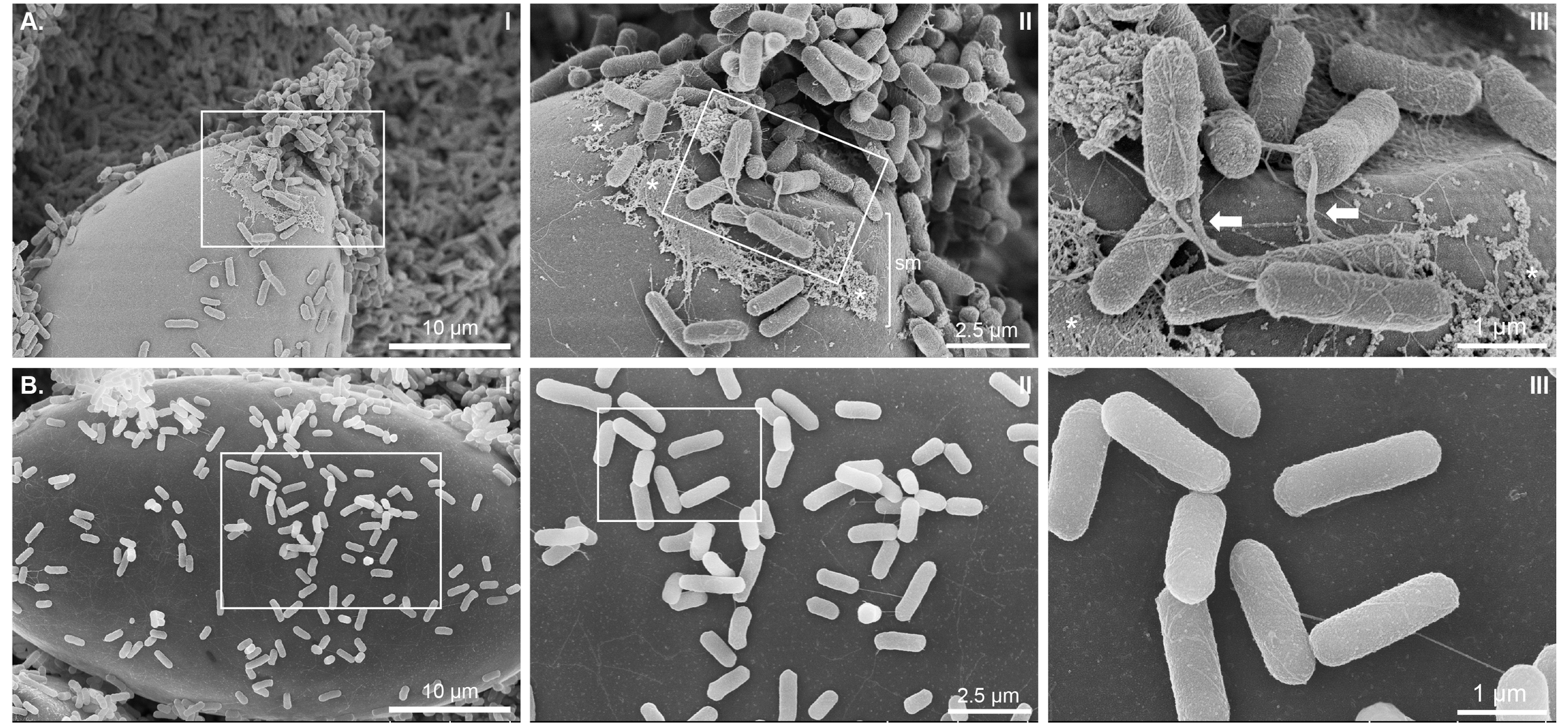

Supplement: S1 Fig — Representative SEM images from T. muris eggs co-cultured with E. coli for 60–90 min at 37°C. Bacteria attached to the eggshell surface are shown: (A) at the polar plug and egg collar; and (B) between the two collars. Insets II and III show increased magnification views of bacteria that bind via fimbriae (in A) to surface material (sm, asterisks) covering the polar plugs and egg collars. The sm is absent between the two collars (B) and bacteria attaching to these areas have no fimbriae. (TIF) [file ppat.1012502.s001.tif]

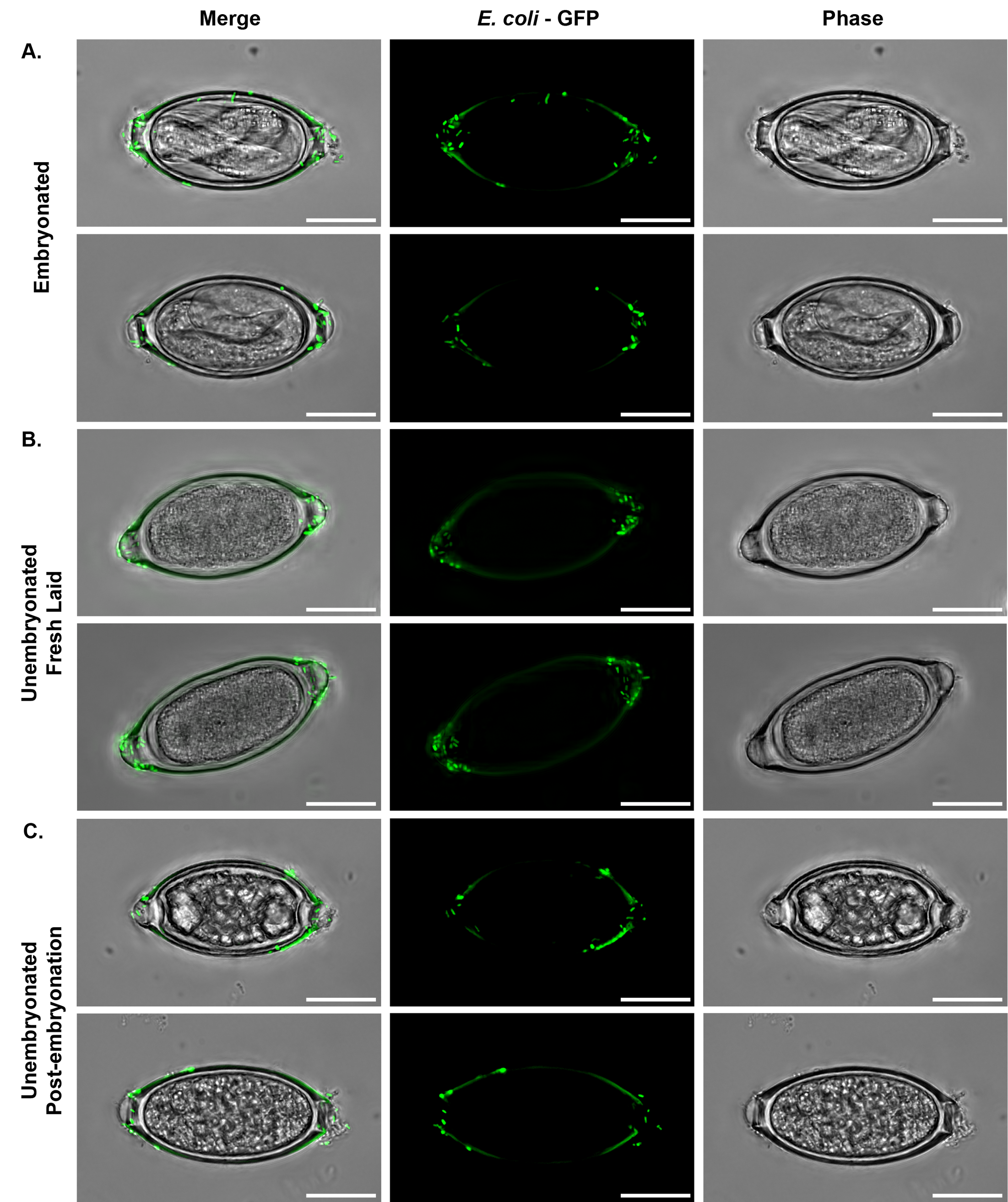

Supplement: S2 Fig — Representative extended depth of field projections of phase contrast and green fluorescent microscopy z-stack images from T. muris eggs—A. embryonated, B. un-embryonated, freshly laid, and C. un-embryonated, after embryonation period—and co-cultured with GFP-expressing Escherichia coli for 75 min at 37°C (scale bars = 20 μm). (TIF) [file ppat.1012502.s002.tif]
