# Supplementary material for: Hatching of whipworm eggs induced by bacterial contact is serine-protease dependent
Source: PLoS Pathog. 2025 Jan 27;21(1):e1012502. doi: 10.1371/journal.ppat.1012502 (PMC11819529; doi:10.1371/journal.ppat.1012502)
Supplement: Attachment — Submitted filename: 2024, Goulding, et al. Response to Reviewers.pdf [file ppat.1012502.s014.pdf]

Re: Revision of manuscript Ms. PPATHOGENS-D-24-01736 - Hatching of whipworm eggs induced by bacterial contact is serine-protease dependent.

We thank the Reviewers for their extensive and insightful comments on our manuscript (ms. PPATHOGENS-D-24-01736). Please find below our detailed point-by-point revision.

Part I – Summary

Please use this section to discuss strengths/weaknesses of study, novelty/significance, general execution and scholarship.

Reviewer #1: Thank you for the opportunity to review the manuscript titled "Hatching of whipworm eggs induced by bacterial contact is serine-protease dependent." The study presents significant contributions to the mechanisms of whipworm egg hatching. This research is particularly relevant given the widespread impact of *Trichuris* spp. on both human and animal health, especially in neglected areas with limited research funding and increased difficulty in publishing results.

The authors have employed various methodologies, including scanning and transmission electron microscopy, to elucidate the intricate processes underlying whipworm egg hatching. I appreciate the thorough approach taken in this study and look forward to providing constructive feedback that may help enhance the manuscript, particularly regarding the results of the ultrastructure.

Reviewer #2: The manuscript by Goulding et al. is a concise and well-written description of a series of experiments undertaken to better understand the requirement for the successful hatching of eggs of the murine whipworm *Trichuris muris*. This is an important area, as whipworms are a widespread parasite of humans and animals that cause much disease. A better understanding of this crucial aspect of the parasite biology may lead to novel treatment options. Over the past 10-15 years, a series of papers has demonstrated a requirement for bacterial contact at the polar plugs to initiate hatching, and more recently a requirement for worm-derived chitinase has been proposed. However, there are still substantial gaps in our knowledge, and this paper addresses them through a solid combination of electron microscopy, transcriptomics and functional assays. A requirement for serine proteases is convincingly demonstrated, although whether these are from the bacteria or the worm is equivocal (see also below).

This paper makes an important contribution to the parasitology literature and is suitable for publication in PLOS Pathogens.

Reviewer #3: The strength of the manuscript is in the serine-protease dependence findings. The weaknesses in the study have to do with the attribution of bacterial causes to technique artifacts induced by application of drying procedures during preparation for electron microscopy. The artifacts under consideration are amplified in the nematode eggshell in ways that have been known for decades but are not widely recognized by authors analyzing TEM & SEM photos.

I am not qualified to comment on other aspects of the MS.

R: We thank the reviewers for the appreciation of our work and its contribution to the understanding of the mechanisms of whipworm egg hatching. We are grateful for the feedback, based on which we have sought to enhance our manuscript.

Part II – Major Issues: Key Experiments Required for Acceptance

Please use this section to detail the key new experiments or modifications of existing experiments that should be absolutely required to validate study conclusions.

Generally, there should be no more than 3 such required experiments or major modifications for a "Major Revision" recommendation. If more than 3 experiments are necessary to validate the study conclusions, then you are encouraged to recommend "Reject".

Reviewer #1: (No Response)

Reviewer #2: N/A

Reviewer #3: I do not necessarily recommend additional experiments, but do insist on a complete revision of all references to anatomical aspects of nematode eggshells. There are many clumsy errors in interpretation of eggshell effects that must be corrected.

Rather than recommend experiments, I would be willing to meet with the authors over Zoom, and help them to understand the errors of interpretation. However, I would also require that they completely re-read in detail two of the papers they cited; namely Bond & Huffman 2023 and Robertson, et al., 2023. If they had read these papers carefully before writing the egg anatomy and effects prose, most of the mistakes would not have been made. This is very important, and if the authors are not willing to rewrite most of their anatomical interpretations of bacterial effects on Trichuris eggs, I would recommend rejection.

R: Following the advice of Reviewer 3, we have re-read both papers suggested and made our utmost to re-interpret the effects of bacterial co-culture on the *T. muris* eggs, acknowledging the artefacts induced by application of drying procedures during preparation for electron microscopy (lines 349–351). Moreover, we have extensively reviewed all our material and included new SEM and TEM images from untreated *T. muris* eggs (not exposed to bacteria) and those co-cultured with both fimbriated and non-fimbriated bacteria to strengthen our observations and replace figures where artefacts could mislead the readers.

Part III – Minor Issues: Editorial and Data Presentation Modifications

Please use this section for editorial suggestions as well as relatively minor modifications of existing data that would enhance clarity.

Reviewer #1:

<u>Introduction:</u>

Lines 69–70: I suggest that the authors include important references that recovered eggs of T. trichiura in non-human primates, as this provides crucial evidence that it can be a zoonotic disease in some countries. As a zoonosis, this impacts the control of egg environmental contamination (refer to line 83).

Additionally, I recommend including this general idea in the discussion and conclusion. This possibility could highlight the sharing of bacterial species in the human and non-human primate microbiota, presenting a fascinating situation that ties into the evolutionary aspects shared by humans and non-human primates.

References: https://doi.org/10.1016/j.vprsr.2017.11.004; https://doi.org/10.3389/fvets.2020.626120

R: We thank the Reviewer for this suggestion, which indeed highlights the importance of *T. trichiura* infection of non-human primates and its impact on the barriers to eradicate Trichuriasis.

Accordingly, we have modified the sentence in the introduction (line 69) to *“Trichuris trichiura is an obligate parasite of human and non-human primates that cannot be maintained in the laboratory yet”* and included the suggested references. Further, we have modified the final paragraph of the

discussion as follows: *"This new knowledge will steer future research aiming to discover the specific human and non-human primate microbiota-egg interactions controlling the hatching of T. trichiura, which could shed light on the host specificity of Trichuris species and their co-evolution with their hosts. Moreover, armed with this knowledge, it may be possible to develop in vivo and in vitro models of human whipworm infection, and identify new intervention targets based on inducing suicidal hatching or inhibiting the hatching cascade to prevent re-infection."*

Methodology:

- Lines 177, 200, 211, 213, 216: Please specify the exact room temperature in the text.
- Line 216 and 225: Change "osmium fixing" to "post-fixing."
- Line 219: Change "100 ethanol" to "absolute ethanol."

R: Changes have been made following the suggestions of the Reviewer. Specifically, upon the first mention of room temperature, we have indicated it refers to 20–22°C (line 178). Moreover, line 232 (former line 216) now reads '*The eggs were carefully rinsed in SCB three times by centrifugation at 280 g for 5 min at RT before post-fixing and layering with 1% osmium tetroxide (Sigma-Aldrich) and thiocarbohydrazide (Sigma-Aldrich) in SCB over 2 h [43].'* Line 243 (former line 225) now reads '*For transmission electron microscopy (TEM), following primary fixation, the pellet was rinsed carefully with SCB and post-fixed with 1% osmium tetroxide for an hour.'*

Results:

1) Figures 1 and 2: The EM images strongly reinforce the results regarding bacterial interaction. However, I suggest that the authors include the number of samples analyzed/processed, the number of eggs studied, and a quantification (with statistical tests) of the bacteria present on the polar plugs and other parts of the eggshell. This information will significantly enhance the robustness and sustainability of your results.

R: Following the suggestion of the Reviewer, we have included in the Materials and Methods section on "Scanning and transmission electron microscopy" the number of samples processed, and the number of eggs studied for each interaction (lines 240–242 and 256–257).

Below is a representative lower magnification SEM image (Response Figure 1) showing a birds-eye view of vertically oriented eggs, from our co-cultures, anchored on a layer of bacteria and used to study the interactions of bacteria with the polar plug. Even after extensive washes these samples were still bacteria rich, which on the one hand enabled the eggs to sit vertically on the "bacteria-bed" facilitating imaging, but on the other hand precluded the observation of the complete eggs, with almost 95% of the eggs being only half-observable. Therefore, it is very difficult to perform an accurate quantification of the bacteria present on the polar plugs vs other parts of the eggshell. Furthermore, in samples where we can visualise the complete egg, quantification would not be accurate as SEM images show a lateral, rather than a 3D, view of the egg so bacteria cannot be counted around the entire volume.

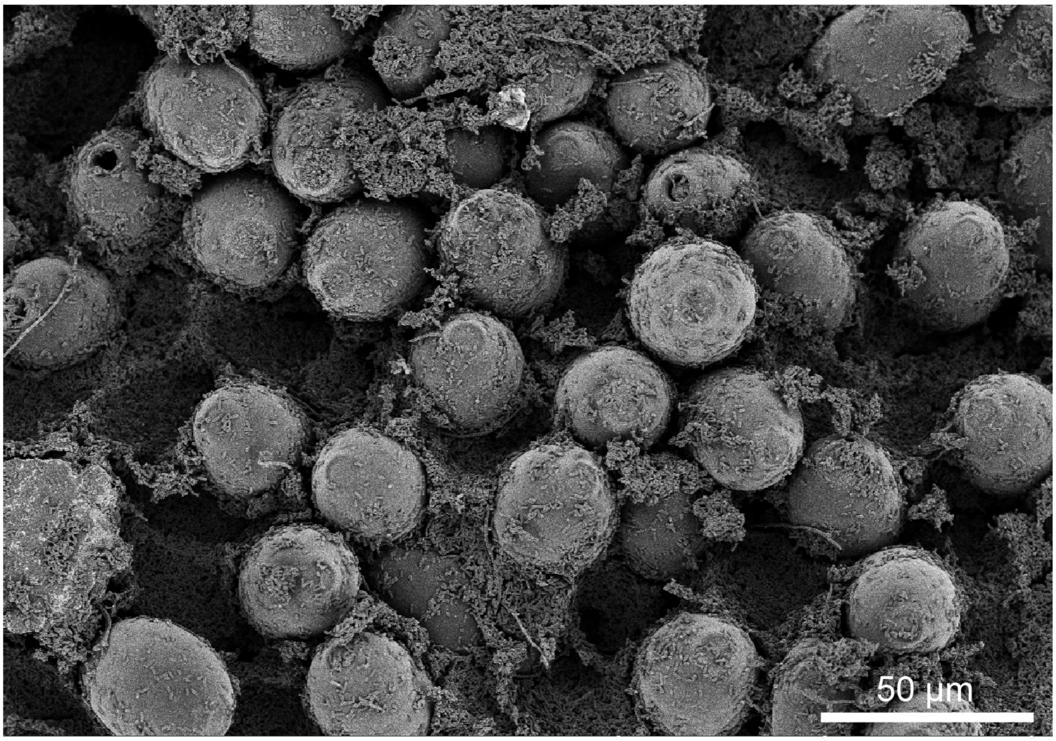

Response Figure 1. Scanning electron microscopy of *T. muris* eggs co-cultured with *Escherichia coli*. Representative low magnification SEM image of *T. muris* eggs co-cultured for 90 min at 37°C with *E. coli*.

Quantification of TEM images presents even more challenges as we are only observing an ultra-thin section of the plug in each image. Therefore, to provide further evidence of our observations on the interactions of fimbriated and non-fimbriated bacteria and *T. muris* eggs, we have assembled a new Figure 2, and gathered further examples, shown below (Response Figure 2).

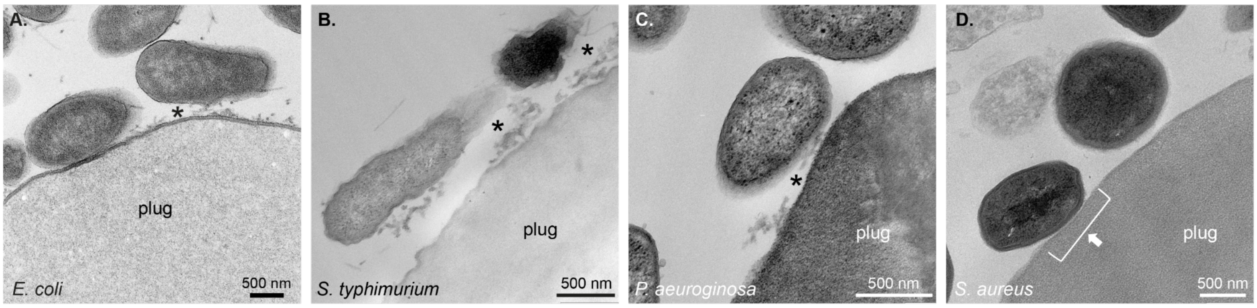

Response Figure 2. Interactions of fimbriated and non-fimbriated bacteria with the *Pellicula Ovi* of *Trichuris muris* eggs polar plugs during the induction phase of the hatching cascade.
Representative TEM images from *T. muris* eggs co-cultured for 60-90 min at 37°C with: **A.** *Escherichia coli,* **B.** *Salmonella typhimurium*, **C.** *Pseudomonas aeruginosa,* and **D.** *Staphylococcus aureus*, showing bacteria in contact with *Pellicula Ovi* (PO) of the *T. muris* egg polar plug. Note bacterial projections (asterisks) anchoring the bacteria to the PO in **A–C**, while in **D.** the cell wall from *S. aureus* is in direct contact with the plug forming a shallow cup-like indentation zone on the PO surface.

2) Figure 2A: Please verify the blue line color of the arrow.

R: This figure is no longer included in the manuscript.

3) Figure 3B: [are the interactions of bacteria seen by TEM with the polar plug real/specific?]

…the bacteria present at the base of the plug (indicated near PO in the figure) may not necessarily be present deeper within the plug; this is a two-dimensional image that might create an illusion of a three-dimensional structure where the bacteria could be positioned more laterally of the plug.

R: While we do agree with the Reviewer on this possibility, each bacteria-polar plug interaction is clearly shown first by SEM in the manuscript (Figures 1 and 2) and then the details are explored by TEM (Figures 2–4).

4) Figure 3B: [presence of artefacts due to TEM preparation, including sinking of the polar plug]

...the authors should be more cautious in the description of figure 3B. The contour of the bacteria outside the halo in the polar plug region, having less electron density, strongly suggests that a material was extracted during ultramicrotomy, which is a common artefact in this process.

In summary, I recommend that the authors carefully interpret these results, particularly when describing: "... the PO with sinking and floccular disruption along the full depth of the plug..." and "Degradation proceeds along the full depth of the plug, both with visible sinking and floccular appearance of the PO..."

R: We are grateful to Reviewers 1 and 3 for their carefully articulated concerns about EM-sample preparation methods causing artefacts that led us to misinterpret results in former Figure 3B. We agree that what we described as a sinking appearance of the polar plug and the clear halo above the plug region in this figure were incorrectly interpreted as an inward disintegration of the plug caused by bacteria from the outside.

We have extensively reviewed our materials in light of Reviewer 1 comments and the further extensive comments from Reviewer 3. The first point to stress is that the sinking effects are specific to bacteria-treated samples. Artefacts in Appleton & White, 1989 and Preston & Jenkins, 1985 and extensively discussed by Bond & Huffman, 2023 were solely due to shrinkage of the plug and do not result in a concave appearance of the plug. In contrast, we have captured an advanced stage of degradation of the polar plug, which is specific to bacteria-treated samples, coupled with the effects of the drying protocols used for TEM-sample preparation.

Our observations of the polar plug collapsing inside the polar canal were, therefore, not directly due to bacteria digesting the plug, but were due to bacteria weakening it and making it more prone to damage/collapse during TEM-sample embedding. Consistent with Robertson et al, 2023, we were unable to prepare TEM samples of whole intact eggs that were not exposed to bacteria (we only captured the polar plugs and eggshell, but not the larva), providing further evidence that bacteria are important to change the permeability of the egg plugs, enabling the downstream TEM preparation steps. Further supporting the idea of a weakened plug that subsequently collapses, we now include an image of an egg at a slightly earlier stage of polar plug degradation, where disintegration of the inner layer of the polar plug (ILPP) can be seen to be starting. This image (Figure 4B; lines 409–411) now replaces the previous Figure 3B.

Thanks to the concerns of the Reviewers, we believe we now have a more accurate interpretation for former Figure 3B. Nevertheless, we have decided to remove the original image (and replace it with a more informative one), to avoid potential misinterpretation by others.

Discussion:

Lines 600-606: It is challenging to compare results obtained by other authors using fluorescence microscopy with GFP-marked bacteria in hydrated conditions to your SEM results obtained in dried samples. Various factors beyond the biological inference could have influenced the adhesion of bacteria to the polar plugs, especially after all the steps in the conventional scanning electron microscopy protocol (including chemical fixation, post-fixation, washing steps, dehydration, and

critical point drying). I recommend either removing this comparison or discussing the possible influences of these parameters on the results.

R: We thank the Reviewer for pointing out the potential differences when comparing SEM and fluorescence microscopy. However, our novel findings using GFP-expressing *E. coli* in both un-embryonated and embryonated *T. muris* eggs (new Supplementary Figure 2), clearly show that bacteria bind both polar plugs and collars in similar numbers (even after extensive washes to remove bacterial excess), results consistent with our SEM observations. We have therefore modified our discussion in lines 645-650 including our new fluorescence microscopy results.

Reviewer #2:

I have a few comments for the authors to consider:

1) Figure 1 – how many weeks of embryonation did the eggs undergo before being used for EM imaging of bacterial attachment?

R: Eggs used in EM experiments were all post 8 weeks of embryonation; after this time, eggs are moved from RT to 4°C, where they are stored for months-years without compromising their *in vitro* hatching potential and *in vivo* infectivity. We have added a line (180) in our Materials and Methods section to clarify this: *"Upon completion of embryonation, eggs were long-term stored at 4°C, where they maintain their in vitro hatching potential and in vivo infectivity."*

Did the authors check whether the stage of embryonation affects bacterial adhesion?

R: No, we did not investigate how embryonation affects bacterial adhesion. Our focus was the transcriptomic changes during egg development that are associated with hatching readiness.

To tackle the Reviewer's question, we have performed a new experiment where we compared binding of GFP-expressing *E. coli* to un-embryonated eggs, freshly laid by *T. muris* females, with that of fully embryonated eggs and those of un-embryonated eggs from the same batch. Interestingly, we found that bacteria bound to both polar plugs and collars of *T. muris* independently of their level of embryonation (new Supplementary Figure 2, described in lines 516-520). We thank the Reviewer for this suggestion, which has uncovered novel insights into the bacteria-egg interactions that mediate *T. muris* hatching.

2) The authors show clearly that serine proteases are important for hatching, as treatment of eggs with either inhibitor cocktails or specific serine protease inhibitors (Pefabloc) ablates hatching, whereas inhibition of other protease inhibitors (e.g. E64) have no effect. However, the manuscript lacks clarity regarding the source of these serine proteases. On page 20, the authors propose that, because inhibitor treatment is reversible, it must be acting on bacterial proteases outside the egg and thus bacteria-derived enzymes are key for initiating egg hatching. However, further analyses with RNAseq on Trichuris L1 suggest that parasite proteases are upregulated concomitant to hatching 'readiness', and thus likely play a role as well. However, the importance of these parasite-derived molecules is difficult to ascertain. If they played a key role in the hatching process, then at least a degree of hatching would presumably be observed in the absence of bacteria. It could be speculated that the upregulation of these genes in the parasite plays no role in hatching per se, and is instead an adaptation of the parasite to be 'primed' to deal with the external environment once hatching is complete.

I appreciate it would be difficult to tease apart these effects – is it possible, for instance, to microinject serine proteases inhibitors into the egg to affect the parasite enzymes whilst leaving the bacterial activity intact on the egg surface?

R: The Reviewer raises a good point but also highlights a critical issue for the field, where a lack of molecular tools, such as transgenesis, mean that it is not possible to ablate expression and

determine the function of these proteases within the larva. Likewise, the proposal of the Reviewer to microinject serine protease inhibitors into the egg is not feasible. Working together with colleagues that regularly perform microinjection as part of their day-to-day work, we have so-far failed to develop a technique that successfully allows microinjection into *T. muris* eggs.

Based on our results, we hypothesised in the discussion, lines 705-710, "*that binding of bacteria to the polar plug results in secretion of bacterial proteases that initiate the disintegration of the polar plug from the outside. This initial degradation could result in endosmosis that may rehydrate and activate the larvae [9,63] (increased larval activity was evidenced by expression of the glycolysis pathway) to secrete serine proteases that can finalise the degradation of the polar plug from within the egg".* According to this model, without the initial degradation of the polar plug by the bacteria, hatching is not possible as the larva will not be rehydrated and activated. This supports the observation that eggs do not hatch spontaneously. However, we do agree with the Reviewer about the possibility that serine-proteases from the larvae could play no role in hatching and have modified the discussion to include this caveat in lines 713-716 *"Nonetheless, it is also possible that the upregulation of these genes in the parasite plays no role in hatching per se, and is instead an adaptation of the parasite to be 'primed' to deal with the external environment or invade the intestinal epithelium once the larvae emerges from the egg"*.

3) Finally – a general comment – whilst I don't doubt the importance of gut bacteria in the infectious process of whipworms, it is likely to be complex. There are older papers showing that infection seems to proceed normally in antibiotic treated mice infected with T. muris - Schopf et al. 2002, J. immuol, or pigs infected with T. suis – Mansfield and Urban 1996, Vet Immunol. Immunopathol. These papers are not often cited in this context.

R: The capacity of antibiotics to compromise infection very much depends on the specific antibiotic used, the dosage and timing. Even where antibiotics have been administered and shown to affect worm burden, inhibition is far from complete and some worms still establish. In the publications mentioned by the Reviewer, neomycin sulphate, or erythromycin lincomycin/spectinomycin were used. As administered, these would not have major effects on reducing the intestinal microbiota. Ultimately, the lack of *T. muris* hatching and infection of germ-free mice (White, *et al*. 2018) has demonstrated the critical role of the microbiota on whipworm establishment in their host.

Reviewer #3:

I have attached the copy of the PDF I reviewed, which contains several minor points of grammar and word choice and some references to anatomical errors.

We thank the Reviewer for providing their copy of the reviewed manuscript. We have incorporated their suggestions throughout the text, including an adjustment to ensure that it reflects the speculative nature of some of the processes that take place during the hatching cascade (lines 96-105).

Major comments directly attached to PDF:

1) Comment about Fig 2D description: In contrast, the S. aureus cell wall directly touched the PO of the egg's polar plug, causing a shallow cup-like indentation (Fig 2D). *"Unless you are seeing these indentations occurring consistently when a coccus is adjacent to the PO, I don't think it is important, and may be spurious."*

R: Our SEM images on Figure 1D supports our TEM observations, with *S. aureus* bacteria making a cup-like indentation at the surface of the *T. muris* egg polar plug. Furthermore, we have now provided additional TEM evidence (see response to Reviewer 1, Comment 1 to Results, Response Figure 2) showing that these indentations do occur consistently when a coccus is adjacent to the PO.

2) [General comment about figure 2]

“There are several artefacts of EM technique in these electron micrographs that the authors are conflating with bacterial activity. Note the crenulated appearance of the PO where it passes over the Outer Layer of the Polar Plug (OLPP). That is due to extreme drying of the otherwise hydrated material of the polar plugs of Trichuris, for which bacteria are not required. You will find this discussed thoroughly in Bond & Huffman, 2023.”

“It is clear that an important aspect of Trichuris egg hatching has been discovered here. However, the authors have not taken enough care to distinguish between random juxtapositioning of bacteria with the polar plugs and random debris, and some of their anatomical conclusions are not yet sound enough to be reported here.”

R: We acknowledge the Reviewers 1 and 3 concerns on sample-preparation artefacts that could be conflated with bacterial activity. We do agree that crenulation due to drying/shrinking was clearly visible in former Figure 2 (current Figure 3). However, the bacterial interactions with the *Pellicula Ovi* (PO) observed by TEM were corroborated by SEM (Figure 1). Because our results really require SEM and TEM evidence (from the same batch of eggs) to be viewed in parallel, we have included a new figure (2) that brings SEM and TEM evidence together.

To further address their concerns about artefacts and juxtapositioning of bacteria with the polar plugs, we have thoroughly re-evaluated our original images as well as processed new material and openly acknowledged the effects of sample preparation for ultrastructural TEM in our results description (lines 349-351). We now present new results in the current Figure 2B showing that *T. muris* eggs that have not been co-cultured with bacteria (i.e. control-untreated eggs). While untreated eggs do show some signs of shrinking, they do not present any “random debris” or fibrillar material binding to their PO. Furthermore, new TEM micrographs of eggs co-cultured with *E. coli* much more clearly show a cross-sectional view of tube-like fimbriae directly beneath and anchoring *E. coli* to the PO’s polar plug (Figure 2D), which are much more visible in our SEM results (Figure 1A, and novel Supplementary Figure 2A and Figure 2C). In addition, we have provided new TEM evidence (see response Reviewer 1, Comment 1 to Results, Response Figure 2) showing fimbriae of *S. typhimurium* and pili of *P. aeruginosa* interacting the egg polar plug, similarly to our SEM findings of Figure 1.

3) [Comment on collapse of the polar plug - Figure 3B]

“collapse of the plug is not degradation caused by bacteria, and the authors should be more careful about making such claims. Note the vacuous space in B with a notch in the top margin of the space. That notch was where the single bacterium (on the PO) formerly was prior to shrinkage of the polar plug material into the polar canal during the drying phase of the specimen prep. The plug contracted back into the polar canal after fixation and drying, leaving the vacuous space behind. When this specimen was fixed, the PO filled that vacuous space and the lone bacterial cell was in the notch.
As can be clearly seen in one of the papers cited by the authors (Robertson, 2023; Fig. 4), the plug does NOT shrink back into the canal in the absence of critical point drying, despite bacterial degradation.
Note also in the imagery of Fig. 2 of this paper how the PO is crenulated over the polar plug but smooth over the chitinous wall. This can occur without bacteria, and the crenulations are caused by shrinkage of the plug bacteria allowing the formerly stretched and turgid PO to suddenly collapse into wrinkles when the pressure previously causing the turgidity to disappear.”

“This "sinking" of the PO is not sinking of the PO. It is simply following the shrinkage of the Outer Layer of the Polar Plug (OLPP) which the authors mislabeled as "zone of striation" and the Inner Layer of the Polar Plug (ILPP) which the authors mislabeled as "zone of granulation." Both of these layers shrink dramatically during drying of specimens during preparation of specimens for TEM sectioning.”

R: As detailed in our response above (see response Reviewer 1, Comment 3 to Results), this Reviewer's comments have been particularly insightful in our re-interpretation of the previous Figure 3B. In short, we agree that the collapse of the plug is not direct degradation caused by the bacteria. In light of the re-interpreted evidence, and the replacement Figure 3B (current Figure 4B) that shows an earlier stage of changes to the plug's properties, we believe that the collapse is a consequence of the bacteria and larva proteases changing the structure/integrity of the plug, manifesting in the plug contracting into the polar canal after dehydration. The Reviewer raised some additional fine-grained remarks about specific anatomical labels on the Figure, which are no longer relevant because that figure has been removed.

4) [Comment on choice of reference]: Consistent with previous works […,22–24], unhatched embryonated *T. muris* eggs showed a PO that continuously covered the polar plug and the CL, and their polar plug presented a **uniform electron density** with no signs of degradation.

"Reference 23 (Beer, 1973) focuses on embryogenesis and larval structure, and is by no means an authority on nematode eggshell structure. That paper provides no TEMs of eggshells; indeed uses the term "vitelline layer" (and mermbrane) to refer to two totally different structures, and authoritatively refers to three thick outer layers, which are artifacts of optical illusions caused by the birefringent properties of chitin. The authors should use more care in selection of anatomical authorities as references in support of their conclusions…"

R: We respectfully note that reference 23 is not Beer, 1973; it is Appleton and White, 1989, and does indeed contain TEMs and SEMs.

5) [Comment on **uniform electron density**]: *"Not all TEMs of all bipolar eggs show the difference between the outer and inner layers of the polar plugs, but the layers are distinct, nonetheless."*

R: We are not quite sure what the Reviewer is suggesting here; we are not discounting the existence of distinct layers, rather, we are simply describing how the plug appears uniform in an egg that has not undergone degradation of the polar plug.
